# In vivo overexpression of synaptogyrin‐3 promotes striatal synaptic dopamine uptake in LRRK2^R1441G^ mutant mouse model of Parkinson's disease

**DOI:** 10.1002/brb3.2886

**Published:** 2023-01-09

**Authors:** Philip Wing‐Lok Ho, Lingfei Li, Hui‐Fang Liu, Zoe Yuen‐Kiu Choi, Eunice Eun Seo Chang, Shirley Yin‐Yu Pang, Yasine Malki, Chi‐Ting Leung, Michelle Hiu‐Wai Kung, David Boyer Ramsden, Shu‐Leong Ho

**Affiliations:** ^1^ Division of Neurology Department of Medicine School of Clinical Medicine University of Hong Kong Hong Kong China; ^2^ Institute of Metabolism and Systems Research University of Birmingham Birmingham UK

**Keywords:** behavioral test, dopamine uptake, LRRK2 mutation, SYNGR3

## Abstract

**Background:**

Leucine‐rich repeat kinase 2 (LRRK2) mutation is a common genetic risk factor of Parkinson's disease (PD). Presynaptic dysfunction is an early pathogenic event associated with dopamine (DA) dysregulation in striatum of the brain. DA uptake activity of DA uptake transporter (DAT) affects synaptic plasticity and motor and non‐motor behavior. Synaptogyrin‐3 (SYNGR3) is part of the synaptogyrin family, especially abundant in brain. Previous in vitro studies demonstrated interaction between SYNGR3 and DAT. Reduced SYNGR3 expression was observed in human PD brains with unclear reasons.

**Methods:**

Here, we further explored whether inducing SYNGR3 expression can influence (i) cellular DA uptake using differentiated human SH‐SY5Y neuronal cells, (ii) striatal synaptosomal DA uptake in a mutant LRRK2^R1441G^ knockin mouse model of PD, and (iii) innate rodent behavior using the marble burying test.

**Results:**

Young LRRK2 mutant mice exhibited significantly lower SYNGR3 levels in striatum compared to age‐matched wild‐type (WT) controls, resembling level in aged WT mice. SYNGR3 is spatially co‐localized with DAT at striatal presynaptic terminals, visualized by immuno‐gold transmission electron microscopy and immunohistochemistry. Their protein–protein interaction was confirmed by co‐immunoprecipitation. Transient overexpression of SYNGR3 in differentiated SH‐SY5Y cells increased cellular DA uptake activity without affecting total DAT levels. Inducing SYNGR3 overexpression by adeno‐associated virus‐7 (AAV7) injection in vivo into striatum increased ex vivo synaptosomal DA uptake in LRRK2 mutant mice and improved their innate marble burying behavior.

**Conclusion:**

Brain SYNGR3 expression may be an important determinant to striatal DA homeostasis and synaptic function. Our preliminary behavioral test showed improved innate behavior after SYNGR3 overexpression in LRRK2 mutant mice, advocating further studies to determine the influence of SYNGR3 in the pathophysiology of DA neurons in PD.

## Graphical abstract

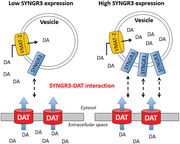

## 1 INTRODUCTION

Parkinson's disease (PD) is the second most common neurodegenerative disorder (Poewe et al., 2017). Its etiology involves a combination of genetic susceptibility, environmental factors, and aging (Pang et al., 2019). Pre‐synaptic dysfunction with impaired dopamine (DA) turnover and oxidative stress is an early pathogenic mechanism which eventually leads to nigrostriatal dopaminergic neurodegeneration (Belluzzi et al., 2012). Such progressive loss of dopaminergic neurons in substantia nigra pars compacta (SNpc) and their projections to the corpus striatum causes striatal DA depletion which manifest as bradykinesia, rigidity and rest tremor in PD (Burke & O'Malley, 2013; Cheng et al., 2010).

SYNGR3 is a member of the synaptogyrin family, especially abundant in brain (Abraham et al., 2011; Belizaire et al., 2004). Among the four homologues (SYNGR1‐4), SYNGR3 is mainly expressed in the central nervous system (CNS) (Kedra et al., 1998). Similar to synaptophysin (SYP), which is a well‐described vesicular marker protein expressed at presynaptic nerve termini, SYNGR3 is localized on synaptic vesicles as a membrane‐spanning structural protein, implicating a role in neurotransmission (Belizaire et al., 2004; Sugita et al., 1999). To elucidate the regulation of *SYNGR3*, we recently carried out an in silico analysis of the 5′‐flanking region of *Syngr3*, where we identified CpG‐rich regions and transcriptional regulatory elements including putative nerve growth factor‐induced clone B (NGFI‐B) response elements (NBRE) that bind nuclear receptor‐related 1 (NURR1) protein (Li et al., 2022). NURR1 is crucial in the development of neuronal stem cells and survival of mature DA neurons (Ramsden et al., 2001). Reduced SYNGR3 expression was reported in PD (Simunovic et al., 2009), Alzheimer's disease (AD) (Saetre et al., 2011), and cancers (Cayre et al., 2007). Similar reduction was also observed in an MPTP mouse model of PD (Miller et al., 2004). These reports suggest a possible role of SYNGR3 in synaptic dysfunction in PD.

DA is a monoamine neurotransmitter involved in several CNS pathways including the nigrostriatal system. DA mediates a wide range of physiological functions including regulation of motor and non‐motor functions (Chaudhuri & Schapira, 2009). During neurotransmission, DA stored in presynaptic vesicles is released into the synaptic cleft where it interacts with dopamine receptors at the postsynaptic terminal (Sulzer et al., 2016). DA in the synaptic cleft is then recycled back into presynaptic termini via dopamine uptake transporter (DAT) and is rapidly repackaged into synaptic vesicles via vesicular monoamine transporter‐2 (VMAT‐2). DA homeostasis is maintained by DA re‐uptake activity primarily mediated by DAT and vesicular packaging (Bu et al., 2021). Mishandling of free DA is associated with auto‐oxidation and resultant generation of reactive oxygen species in synaptic nerve termini (Burbulla et al., 2017), compromising the survival of DA neurons (Puspita et al., 2017). A recent in vitro study showed that SYNGR3 interacts with DAT to facilitate cellular DA uptake process, which was abolished with reserpine, a VMAT‐2 inhibitor (Egana et al., 2009).

LRRK2 mutations represent one of the most common genetic risks of PD (Kluss et al., 2019; Pang et al., 2019). LRRK2‐PD has similar clinical and typical Lewy‐type neuropathological features as sporadic PD. We have developed a colony of mutant LRRK2^R1441G^ knockin mice with a single base substitution resulting in non‐synonymous R1441G mutation in Ras‐of‐complex (Roc) GTPase domain (Liu et al., 2014). These mice are susceptible to reserpine‐induced DA depletion and locomotor deficits (Liu et al., 2014). In this study, we explored whether inducing SYNGR3 expression can influence (i) cellular DA uptake in vitro using a differentiated human neuronal cell line, (ii) striatal DA uptake ex vivo using our mutant LRRK2^R1441G^ knockin mouse model, and (iii) innate rodent behavior using the marble burying behavioral test.

## 2 MATERIALS AND METHODS

### 2.1 Animals

A C57BL/6 mouse colony with complete homozygous knockin of pathogenic LRRK2^R1441G^ knockin mutation (‘‘R1441G’’ mutation in the ROC GTPase domain of LRRK2) was generated as described previously (Liu et al., 2014). These mutant mice were back‐crossed with wild‐type (WT) C57BL/6 for eight generations and maintained under pure C57BL/6N mouse background. All mice were maintained on a 12‐h light/dark cycle, with lights on at 7 a.m. in the Laboratory Animal Unit, University of Hong Kong which has accreditation through the Association for Assessment and Accreditation of Laboratory Animal Care International (AAALAC), with unrestricted access to food and water. All animal procedures were performed in accordance with the National Institute of Health Guide for the Care and Use of Laboratory Animals and were approved by the Committee on the Use of Live Animals in Teaching and Research (CULATR; application No. 4043‐16) of the University of Hong Kong.

### 2.2 Cell culture

Human SH‐SY5Y neuroblastoma cells were obtained from the American Type Culture Collection (ATCC®; CRL‐2266), and cultured in DMEM/F12 supplemented with 10% fetal bovine serum (FBS), 50 μg/ml penicillin and streptomycin at 37°C in a humidified, 5% CO_2_ incubator. Cells were subsequently differentiated by all‐*trans* retinoic acid (RA; 10 μM) for 7 days before experiments.

### 2.3 Overexpression of SYNGR3 in human SH‐SY5Y cells

Human SYNGR3 protein was overexpressed (transfection of expression plasmid) in RA‐differentiated SH‐SY5Y cells. Briefly, the full length human *SYNGR3* cDNA was amplified by PCR with forward primers: 5′‐ATAGGATCCATGGAGGGCGCCTCCTTC‐3′ and reverse primer: 5′‐ATACTCGAGGTAGGCGGGCACCTGGTA‐3′. The PCR products were subcloned into a mammalian expression vector, pcDNA3.1(+) using *BamHI* and *XholI* restriction enzymes, and then heat shock transformed into competent One Shot® OmniMAX™ 2 bacterial cells. The positive sequencing confirmed that plasmid was transfected into SH‐SY5Y cells using Lipofectamine2000. Cells transfected with empty vector, pcDNA3.1(+), were used as controls.

### 2.4 Immunocytochemistry of SYNGR3 and SYP

SYNGR3‐GFP overexpressing SH‐SY5Y cells were seeded at 70% confluence in chamber slides before the immunostaining. Cells were fixed with 4% paraformaldehyde for 10 min at room temperature (RT) and incubated with antibody against synaptophysin (SYP) (1:200, Cell Signaling Technology #D35E4) at 4°C overnight, followed by co‐incubation with Alexa Fluor 546‐conjugated anti‐mouse IgG (1:800) for 1 h. Sections were mounted using antifade mounting medium, and cell images were visualized under confocal microscope (Carl Zeiss LSM 510). Cellular nuclei were counterstained with DAPI.

### 2.5 SDS‐PAGE/Western blot analysis of SYNGR3 levels in transfected SH‐SY5Y cells

Cells were lysed in ice‐cooled 1× RIPA lysis buffer (Cell Signaling Technology) with a protease inhibitor cocktail (Roche). The cell lysates were incubated on ice for 20 min and clarified by centrifugation at 4°C for 15 min at 12,000 × *g*. Protein concentration was determined by the Bradford assay (ThermoFisher™ Scientific, #5000205). The lysate solution was boiled for 5 min at 100°C in 1× denaturing sample buffer (Pierce). Samples containing the same amount of protein were placed in the wells of a 10% polyacrylamide gel (375 mM Tris, 10% Acrylamide/Bis, 0.1% SDS, 0.05% APS and 0.15% TEMED) and electrophoresed in Tris‐Glycine SDS running buffer (25 mM Tris, 190 Mm glycine, and 0.1% SDS; pH 8.3) at 80 V for 30 min followed by 100 V for 90 min. Separated proteins were transferred onto a nitrocellulose membrane by electrophoresis in Tris‐Glycine transfer buffer (25 mM Tris, 190Mm glycine, and 15% methanol; pH 8.3) at 100 V for 2 h. The membrane was blocked with 5% non‐fat skimmed milk in TBS and probed with antibodies against SYNGR3 (1:3000, Santa‐Cruz Biotechnology; #sc‐271046, 26 kD), synaptophysin (SYP) (1:1000, Cell Signaling Technology #D35E4, 38 kD), DAT (1:1000, Santa‐Cruz Biotechnology #sc‐10042, 50 kD), and actin (1:3000, Santa‐Cruz Biotechnology #sc‐1615, 43 kD). For chemiluminescence detection, blots were incubated with HRP‐conjugated secondary antibodies (DAKO) followed by ECL substrate (GE Lifesciences). Immunoblots were scanned and the scanned images were analyzed by the ImageJ software (http://rsbweb.nih.gov/ij/plugins/track/track.html). Levels of cell surface DAT were determined by flow cytometry after whole cell immunostaining using fluorophore‐linked antibody targeting the extracellular loop of DAT (1:500, Santa‐Cruz Biotechnology #sc‐32259). The mean cellular fluorescent intensity was determined by flow cytometry which indicate levels of cell surface DAT.

### 2.6 Extraction of mouse striatal proteins for SDS‐PAGE/Western Blotting

Whole striatum (from 3‐month‐old and 18‐month‐old WT and KI mice) was dissected and homogenized in 1× cold RIPA lysis buffer supplemented with 0.1% SDS (Cell Signaling Technology) with a protease inhibitor cocktail (Roche). The mixture was incubated on ice for 20 min and clarified by centrifugation at 4°C for 15 min at 12,000 × g. Protein concentration was determined by the Bradford assay (ThermoFisher™ Scientific, #5000205). The lysate solution was boiled for 5 min at 80°C in 1× denaturing sample buffer (Pierce). Lysates equal amounts of protein were subjected to Western blot analysis as mentioned above.

### 2.7 Estimation of SYNGR3 protein levels in mouse brain by ELISA

Whole striatum (from 3‐month‐old and 18‐month‐old WT and KI mice) was dissected and freshly homogenized in 1× ice‐cooled PBS supplemented with PMSF and protease inhibitor cocktail (Roche). After lysate clarification by centrifugation at 4°C for 15 min at 12,000 × *g*, lysate protein concentration was determined by Bradford assay (ThermoFisher™ Scientific, #5000205). The mouse SYNGR3 ELISA kit (MBS9327840; MyBiosource®) was a ready‐to‐use quantitative sandwich ELISA. The detection range for SYNGR3 was 3.12–100 ng/ml, with an estimated sensitivity of 1.0 ng/ml. It was based on SYNGR3 antibody‐SYNGR3 antigen interactions (immunosorbency) and an HRP colorimetric detection system to detect SYNGR3 antigen targets in samples. The kit was designed to detect native, not recombinant SYNGR3. The ELISA was performed according to the manufacturer's protocol.

### 2.8 Whole cell DA uptake assay

SH‐SY5Y cells overexpressing SYNGR3 were seeded into 24‐well plates at 70% cell confluency. After 24 h, the medium was removed, and the cells were rinsed with 0.5 ml of pre‐warmed DA uptake buffer (10 mM HEPES, 130 mM NaCl, 1.3 mM KCl, 2.2 mM CaCl_2_, 1.2 mM MgSO_4_, 1.2 mM KH_2_PO_4_, 10 mM glucose, pH 7.4), and then incubated in 0.5 ml of uptake buffer with 5 M DA and 20 nM [3H]‐DA for 10 min at 37°C. The assay was terminated by washing the cells three times with ice‐cold uptake buffer. Then, cells were lysed with 1% sodium dodecyl sulfate (SDS; 300 μl) for 15 min at RT. Radioactivity was measured in a Beckman scintillation counter with UniverSol cocktail. Nonspecific [3H]‐DA uptake was defined as uptake in the presence of 100 μM nomifensine. Specific uptake was defined as total uptake minus non‐specific in the presence of 10 μm nomifensine. Kinetic parameters were determined by nonlinear regression fitting using Prism software (GraphPad™).

### 2.9 Immunohistochemistry of SYNGR3 and DAT in mouse striatum

Mice were anesthetized with pentobarbital and then perfused with trans‐cardiac cold PBS followed by 4% paraformaldehyde (PFA). Whole brain was dissected and post‐fixed at 4°C overnight. Following dehydration and embedding in paraffin wax, sample tissues were coronally sectioned at 8 μm thickness. After antigen retrieval, sections were incubated with anti‐SYNGR3 (1:200, Santa‐Cruz Biotechnology; #sc‐271046) and anti‐DAT (1:1000, Santa‐Cruz Biotechnology #sc‐10042) at 4°C overnight. After subsequent washes with TBST, sections were then incubated with secondary antibodies conjugated with Alexa Flour™ 488 and 594 nm (ThermoFisher Scientific, USA). Positive immuno‐stained slides were examined and photographed under confocal microscope.

### 2.10 Localization of SYNGR3 and DAT in mouse striatum by immuno‐gold TEM

Two‐month‐old male WT mice were deeply anesthetized and perfused transcardially with ice‐cold 0.1 M PBS solution (5 ml; pH 7.4), followed by freshly prepared ice‐cold 2.5% glutaraldehyde and 2% PFA in PBS (5 ml; pH 7.4). The perfused brain was dissected and post‐fixed at 4°C overnight. Dorsal striatum of the brain was sectioned coronally using vibratome into 10 micron slides re‐suspended in ice‐cooled PBS, pH 7.4. In order to enhance antibody penetration, target striatal sections were incubated in PBS containing 2.5% glycerol, 25% sucrose, and 0.05% Triton‐X 100. The sections were blocked in 0.5% BSA in TBS (pH 7.6) for 30 min at RT and incubated with primary antibody (a mixture of rat anti‐DAT and mouse anti‐SYNGR3) for 36 h at 4°C in 0.1% BSA in TBS. Sections were rinsed at least three times with TBS for 5 min and then incubated with gold particle‐conjugated secondary antibody (1:100) at RT for 2 h. After rinsing three times with TBS for 5 min, sections were fixed in 2% glutaraldehyde in 0.1 M PBS at RT for 10 min. Afterward, sections were incubated in 2% osmium tetroxide at 4°C for 60 min, then dehydrated in a series of graded ethanol and propylene oxide solutions, and embedded in epoxy resin. The resin‐embedded material was observed under EM (Philips EM208s transmission electron microscope) at magnification of 6.5 × 10^4^.

### 2.11 Protein–protein interaction between SYNGR3 and DAT by immunoprecipitation

Whole striatum from 3‐month‐old WT mice was dissected and suspended in 1× ice‐cooled PBS supplemented with PMSF and protease inhibitor cocktail (Roche), followed by sonication for 30 s three times. The resultant lysates were clarified for 30 min at 10,000 × *g* at 4°C. Any endogenous immunoglobulin was removed from the striatal lysate (1 mg per sample) by incubation with Protein A Sepharose beads (EZview™ Red Protein A Affinity Gel, Sigma) overnight at 4°C. The cleared lysate was incubated with either anti‐SYNGR3 or anti‐DAT antibody for overnight at 4°C under gentle agitation, followed by addition of 50 μl of Protein A‐conjugated agarose beads. The lysate‐beads mixture was subjected to rotary agitation for 2 h. The protein‐beads complex was washed three times with PBS. The supernatant was carefully removed. The immunoprecipitated protein was eluted from the beads by boiling in 50 μl sample buffer (Pierce™ Lane Marker Reducing Sample Buffer, ThermoFisher Scientific™) for 10 min. The resultant eluent was analyzed by SDS‐PAGE/Western blotting.

### 2.12 Stereotaxic injection of AAV7‐SynI‐mSYNGR3 into mouse brain dorsal striatum

Dorsal striatum is enriched with nerve termini projecting from DA neurons in the SNpc. Mouse SYNGR3 (NCBI: NM_011522.3) was overexpressed in LRRK2^R1441G^ mutant mouse striatum using a neuronal‐specific Synapsin‐I (SynI) promoter driven adeno‐associated virus serotype 7 (AAV7) encoding the mouse SYNGR3 gene. SynI gene promoter was used to drive expression of mouse SYNGR3 because this is a well‐characterized gene promoter which confers neuron‐specific transgene expression in adult rodent brain over a long period of time (Kugler et al., 2003). The AAV expression construct (pAAV2/7) (Fisher et al., 1997) as cloned to incorporate human SynI promoter and mouse SYNGR3 expression transcript (*pAAV‐SynI‐mSYNGR3*) and verified before the virus was synthesized and purified commercially by SignaGen® Laboratory (MD, USA). Briefly, three plasmids were used for AAV production, including (1) AAV *cis*‐plasmids containing the vector genome, (2) AAV *trans*‐plasmids containing *rep* and *cap* genes, and (3) adenovirus helper plasmids encoding essential adenovirus functions. Plasmid identity assay was performed by SignaGen® Laboratory (MD, USA), including validation sequencing of each functional DNA element and the correct restriction pattern. For AAV *cis*‐plasmid, these elements include the 5′ and 3′ inverted terminal repeats (ITR), mouse *SYNGR3* cDNA, SynI promoter, and polyadenylation (poly‐A) sequence. For *trans*‐plasmids, the presence of the correct *cap* gene was verified by full length sequencing. The recombinant AAV was purified via two rounds of CsCl ultra‐centrifugation followed by desalting and reconstitution in sterile PBS supplemented with pluronic F‐68 at 0.001%. Viral gene copy number of AAV vector as a measure of AAV particles with full genome content was validated by real‐time PCR. Endotoxin assay was carried out using standardized Limulus amebocyte lysate (LAL) gel‐clot method.

Under sterile conditions, mice were anesthetized with ketamine/xylazine and secured in a stereotaxic frame (Figure 4a), a hole to fit the size of the injection needle was drilled on the surface of the skull on one side, and the injection was done with 1 μl of AAV7 solution per brain region using an injection syringe (Hamilton) to deliver at a constant volume of 0.1 μl/min. The needle was left in place for 10 min after each injection to minimize upward flow of viral solution after raising the needle. The brain coordinates to target dorsal striatum were as follows: AP: 0.86 mm; ML: −1.6 mm; DV: −3.8 mm. Control mice were injected with AAV7 expressing green fluorescent protein (GFP) with similar titer to demonstrate viral transduction efficiency. Successful AAV7 infection and overexpression of SYNGR3 was confirmed by immunohistochemistry and Western blotting.

### 2.13 Synaptosomal DA uptake assay in mouse striatum

Mice were anesthetized and sacrificed by decapitation. Left and right striata of the brain were dissected separately and ground using a Dounce homogenizer in ice‐cold lysis buffer (0.32 M sucrose, 0.01 M HEPES, pH 7.4). The striatal lysates were centrifuged with 1000 × *g* at 4°C for 10 min. The supernatants were collected and transferred into a new tube for centrifugation with 10,000 × *g* at 4°C for 20 min. The supernatants were discarded and the synaptosome pellets were resuspended with ice‐cold DA uptake buffer. The synaptosomal [^3^H]‐DA uptake was assayed as previously described (Liu et al., 2014). Briefly, synaptosomes from striatum were isolated and resuspended in Krebs–Ringer buffer (120 mM NaCl; 4.8 mM KCl; 1.3 mM CaCl_2_; 1.2 mM MgSO_4_; 1.2 mM KH_2_PO_4_; 25 mM NaHCO_3_; 6 mM glucose; pH 7.6) with 0.1 μM [3H]‐DA for 5 min at 37°C. DA uptake was stopped by addition of cold Krebs–Ringer buffer (200 μl). The reaction solution was then filtered using a UniFilter®−96 GF/C filter. After washing and drying of the filter, [3H] radioactivity (count per minute, cpm) was measured using a TopCount® NXTTM microplate scintillation and luminescence counter (Packard). Non‐specific uptake was determined in the presence of 10 μM nomifensine (DAT inhibitor). Specific [3H]‐DA uptake was calculated as the difference between [3H] radioactivity in tested sample and paired negative control. Each treatment group was run in duplicate.

### 2.14 Innate marble burying test

Young (3‐month‐old) male WT and LRRK2^R1441G^ mutant mice of similar age from different litters were evenly assigned into different treatment groups to avoid confounding litter effects. Mice receiving stereotaxic injection of either AAV7‐SYNGR3 or AAV7‐GFP were maintained for 3 months and then were subjected to the marble burying test to assess their innate behavior. The marbles used were multi‐colored and 15 mm in diameter. A total of 15 marbles were evenly distributed on the bedding inside the cage in form of a 5 × 3 matrix. Each mouse was transferred to a new cage to perform marble burying test for 30 min. Cages were filled with bedding consisting of aspen wood chips in a layer that was ∼5 cm deep. Behavioral sessions were conducted under similar conditions with reference to published protocol (Njung'e & Handley, 1991). The number of marbles which were two‐thirds covered by the bedding were counted. All the behavioral tests were performed under the same experimental condition on the same day and time to minimize confounding bias.

### 2.15 Statistical analyses

All experiments were performed based on sufficient number of independent trials to achieve statistical significance as indicated in figure legends. Results were expressed as means ± SEM. Conclusions were drawn based on statistical analyses using GraphPad™ PRISM software (GraphPad Inc., CA, USA). Statistical difference between independent groups was assessed by unpaired Mann–Whitney non‐parametric analysis. Comparison between measurements in left and right brain of the same mouse was assessed by paired *t*‐test. Group comparisons were considered significant with *p*‐value was less than .05*p* < .

## 3 RESULTS

### 3.1 SYNGR3 protein expression was reduced in striatum of young LRRK2^R1441G^ mutant mice

**FIGURE 1:**
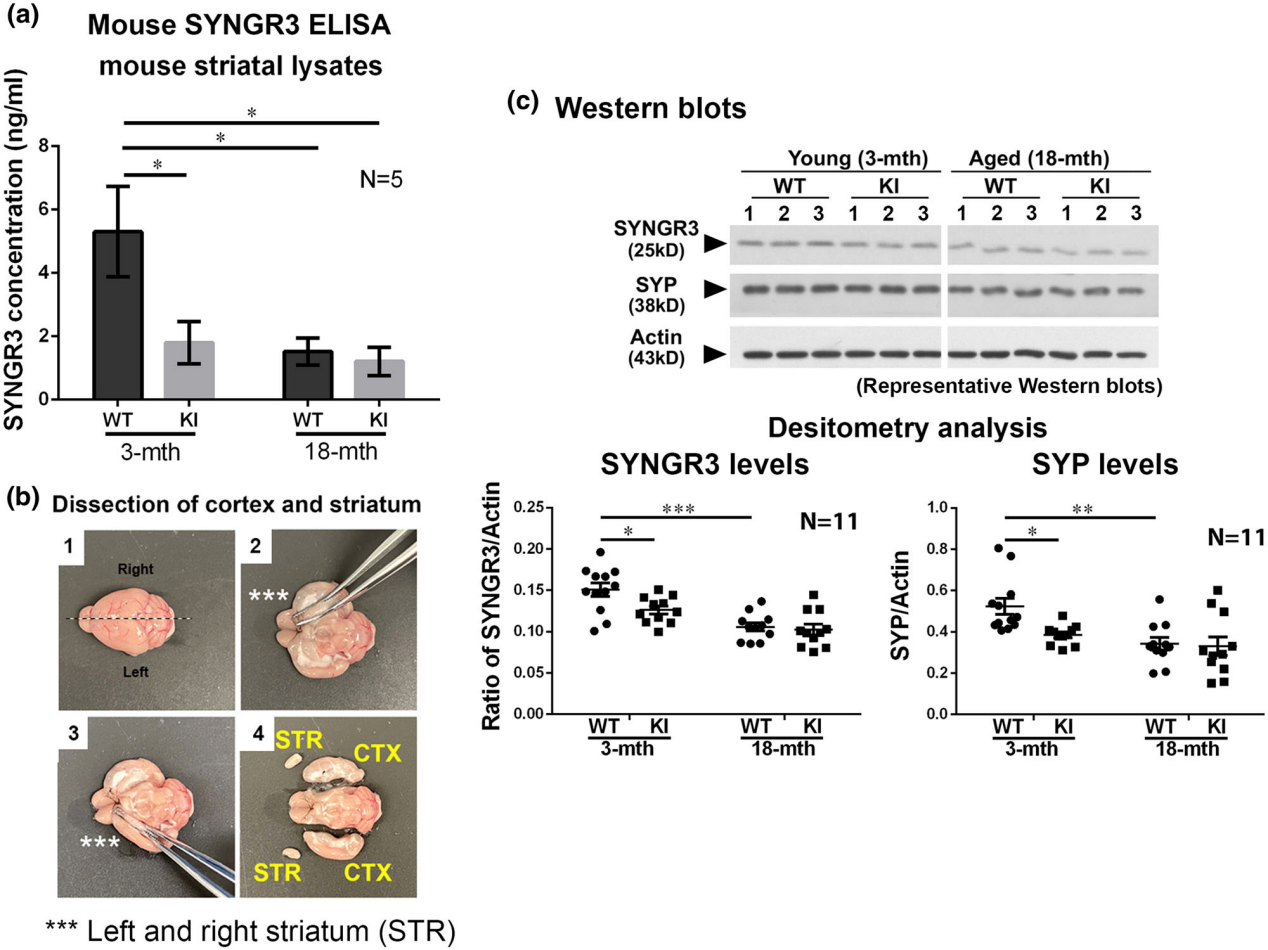
Expression levels of SYNGR3 and synaptophysin (SYP) in striatum lysates from 3‐month‐old and 18‐month‐old wild‐type (WT) and LRRK2^R1441G^ mutant mice. (a) Quantification of SYNGR3 content in whole striatal lysates using a commercial ELISA based on a standard curve developed with a serial dilution of recombinant SYNGR3 protein standards supplied by the kit. (b) Illustrations of mouse brain dissection and isolation of striatum (STR) and cortex (CTX). (c) Representative Western blots of SYNGR3 and SYP in total striatal lysates. Densitometry analysis showed that SYNGR3 and SYP were significantly reduced in young LRRK2 mutant mice compared to WT control. Data are expressed as means ± SEM. (N = 11). *p < .05, **p < .01, and ***p < .001 represent statistical significance between groups by Mann–Whitney (unpaired, nonparametric) test. Abbreviation: KI, knockin

The amount of SYNGR3 in freshly dissected whole striatum from young and aged WT and LRRK2^R1441G^ mutant mice was quantified and compared using mouse SYNGR3 ELISA, based on a standard curve developed from a serial dilution of recombinant mouse SYNGR3 protein standards supplied by the kit (Figure 1a–b). Absolute quantification of SYNGR3 protein content revealed that young LRRK2 mutant mice have significantly lower SYNGR3 levels in the striatum compared with that of age‐matched WT animals (*p* < .05; N = 5) (Figure 1a). The aging effect on SYNGR3 levels was also observed between young and aged WT mice (*p* < .05; N = 5). This difference in striatal SYNGR3 levels was not observed between aged WT and mutant mice.

To verify the ELISA results, we also compared the expression of SYNGR3 and SYP protein in young and aged WT and mutant mouse striatal lysates using Western blotting. Striatal protein lysates of young (N = 11) and aged (N = 11) WT and mutant mice were resolved and subjected to SDS‐PAGE/Western blotting. A band at ∼25 kDa corresponding to SYNGR3 (25 kDa) was immuno‐detected in Western blots of all striatal samples. SYP (∼38 kDa) and actin (∼43 kDa) of each striatal sample were also detected (Figure 1c). Similar to the ELISA results, there was significantly less striatal SYNGR3 in young LRRK2 mutant mice compared with the corresponding level in their age‐ and sex‐matched WT controls (young mutant vs. WT: −16.4%, *p* < .05, N = 11) (Figure 1c). However, levels of SYNGR3 were not significantly different between aged WT and aged mutant striata. An age‐related difference in SYNGR3 was observed in WT mice (aged WT vs. young WT: −26.94%, *p* < .01; N = 11), indicating SYNGR3 levels changed with age. Furthermore, striatal SYP levels were lower in young mutant mice compared with WT controls (young mutant vs. WT: −26.5%, *p* < .01; N ≥ 11), suggesting reduced number of synaptic vesicles in the mutant striatum. Decline in the amount of SYP was also observed with age in WT mice (aged WT vs. young WT: −34.6%, *p* < .01; N = 11). However, striatal SYP levels of aged WT mice compared with those found in age‐matched mutant mice were similar (Figure 1c).

### 3.2 SYNGR3 co‐localized and interacted with DAT in mouse striatum

**FIGURE 2:**
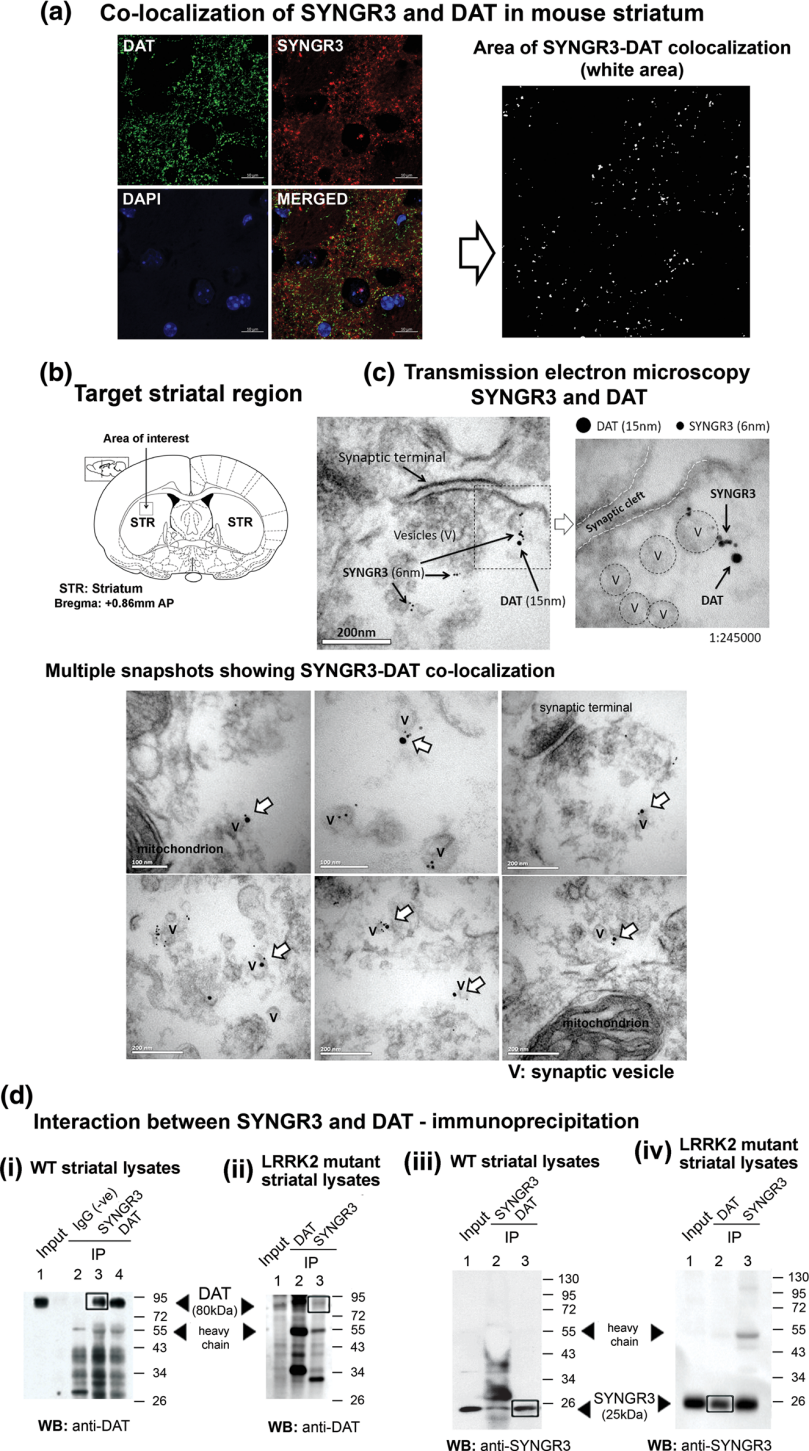
SYNGR3 co‐localizes and interacts with dopamine uptake transporter (DAT) in striatum. (a) Immunohistochemistry showed co‐localization of SYNGR3 (red) and DAT (green) in dorsal striatal region of mouse brain. Area of SYNGR3‐DAT colocalization was identified using Adobe Photoshop™, which were shown as white puncta in the right panel. (b and c) Immunogold staining under transmission electron microscopy (TEM) demonstrated spatial proximity between SYNGR3 and DAT in striatum. Multiple snapshots show SYNGR3‐DAT co‐localization in striatal pre‐synaptic termini; magnification: 1:245,000. (d) Immunoprecipitation of DAT using anti‐DAT antibody resulted in co‐precipitation of SYNGR3 from wild‐type (WT) (i) and LRRK2^R1441G^ mutant (ii) mouse brain striatal lysates, and (ii, iv) vice versa. Target bands are highlighted by the rectangular boxes.

Immuno‐gold transmission electron microscopy (TEM) and immunohistochemistry of SYNGR3 and DAT were performed to investigate the co‐localization between SYNGR3 and DAT in mouse striatum. Immunostaining of SYNGR3 and DAT in mouse brain striatal sections revealed a high degree of co‐localization between these two proteins in the striatum (Figure 2a). To further demonstrate the co‐localization of SYNGR3 and DAT in the brain, these two proteins were separately labeled using specific antibodies conjugated with different sizes of gold particles (6 nm for SYNGR3; 15 nm for DAT). Under TEM, a portion of total SYNGR3 protein was shown to co‐localize with DAT in striatal nerve terminals as shown by the close proximity between labeled SYNGR3 and DAT proteins (Figure 2b). Multiple TEM photomicrographs demonstrated co‐localization of SYNGR3 and DAT on synaptic vesicle surface (Figure 2c).

In additional to the spatial proximity between SYNGR3 and DAT, protein binding between SYNGR3 and DAT was assessed by co‐immunoprecipitation using total striatal lysates from young WT mice. In the resultant pull‐down lysates incubated with anti‐SYNGR3 antibody as the capture antibody, DAT was detected (80 kDa band) (Figure 2d(i)). Simultaneously, we detected the presence of SYNGR3 (25 kDa band) in the resultant pull‐down incubated with anti‐DAT antibody as the capture antibody (Figure 2d(iii)). Similar immunoprecipitation experiments were repeated using LRRK2 mutant mouse striatal lysates, which showed similar interaction between SYNGR3 and DAT (Figure 2d(ii, iv)). These results confirmed direct physical interaction between SYNGR3 and DAT protein in the both WT and LRRK2 mutant brain (Egana et al., 2009).

### 3.3 Transient overexpression of SYNGR3 promoted DA uptake in human differentiated SH‐SY5Y cells

**FIGURE 3:**
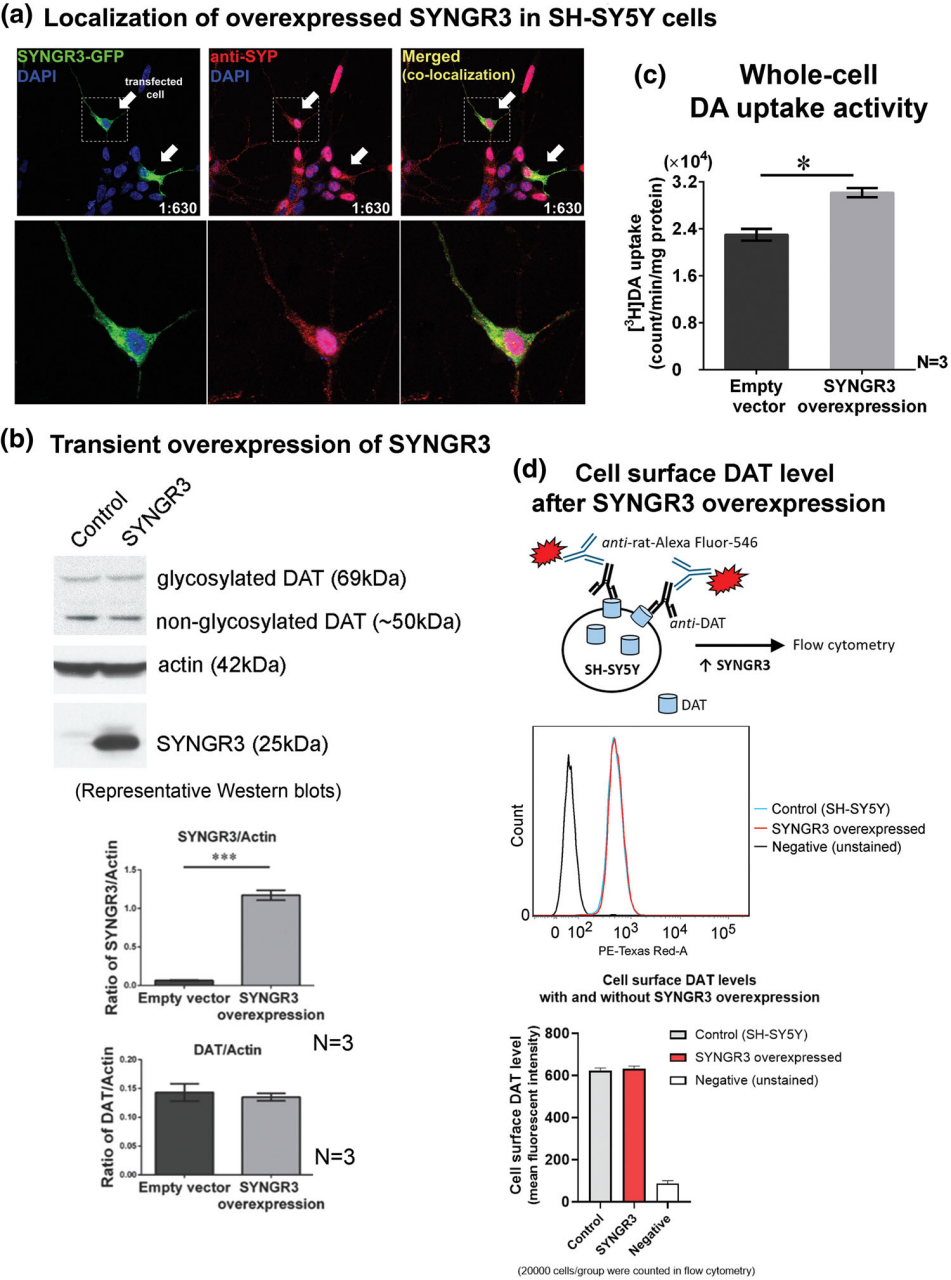
Overexpression of SYNGR3 increased cellular dopamine (DA) uptake in human SH‐SY5Y neuroblastoma cells. (a) Immunocytochemistry showing that SYNGR3 tagged with green fluorescent protein (GFP) (green) was overexpressed and co‐localized with synaptophysin (SYP; synaptic vesicle marker protein; red) in human SH‐SY5Y cells; magnification: 1:630. The lower panels showed magnified views of transfected cells in the dashed box. (b) Western blot analysis of total cell lysates after transient overexpression of SYNGR3 compared to empty‐vector controls. (c) Overexpression of SYNGR3 in SH‐SY5Y cells caused significant increase in total cellular [3H]‐DA uptake activity compared with empty‐vector controls. (d) Flow cytometry assay after whole cell immunostaining of cell surface DAT. Mean cellular fluorescent intensity was calculated based on measurements of 20,000 cells in each group. SYNGR3 overexpression did not affect levels of cell surface DAT. *p < .05, and ***p < .001 represent statistical significance between groups by unpaired Student's *t*‐test.

To determine whether SYNGR3 expression affected DAT activity, human SYNGR3 protein was overexpressed in retinoic acid (RA)‐differentiated SH‐SY5Y cells prior to whole‐cell (3H]‐DA uptake assay. To ensure correct localization of SYNGR3 expression, cells were transiently transfected with plasmid overexpressing green fluorescent protein (GFP)‐tagged SYNGR3 (green) (Figure 3a). The transfected cells were subsequently stained for SYP, a marker protein of synaptic vesicles (red). Merged images under fluorescence microscopy showed co‐localization of SYNGR3 and SYP indicating SYNGR3 was overexpressed in the synaptic vesicles (Figure 3a).

To examine the effect of SYNGR3 expression on cellular DA uptake, SH‐SY5Y cells were transfected with an expression plasmid encoding untagged SYNGR3 [pcDNA3.1(+)‐*h*SYNGR3] or empty plasmid [pcDNA3.1(+)] as controls. Total cellular SYNGR3 protein levels were significantly increased by 120% compared to empty‐vector controls (*p* < .001; N = 3) (Figure 3b). SYNGR3 overexpression in these cells had no significant effect on the total cellular DAT protein levels, including both glycosylated and non‐glycosylated DAT (Figure 3b). Total cellular [3H]‐DA uptake activity was significantly increased by 23% (*p* < .05, N = 3) in cells overexpressing SYNGR3 relative to empty‐vector control cells (Figure 3c). These results indicate that SYNGR3 expression facilitates total cellular DA uptake in human neuronal cells. Furthermore, to determine whether increased DA uptake after SYNGR3 overexpression was due to changes of DAT level on the cell surface, transfected cells were stained by a fluorophore‐linked antibody targeting the extracellular loop of DAT (Figure 3d). Flow cytometry measurements showed that the mean cellular intensity of fluorescent‐labeled cell surface DAT was similar between SYNGR3‐overexpressing and vector control cells (Figure 3d), indicating that SYNGR3 overexpression did not affect the availability of cell surface DAT.

### 3.4 Adeno‐associated virus‐mediated SYNGR3 overexpression in mouse brain striatum increased DA uptake

**FIGURE 4:**
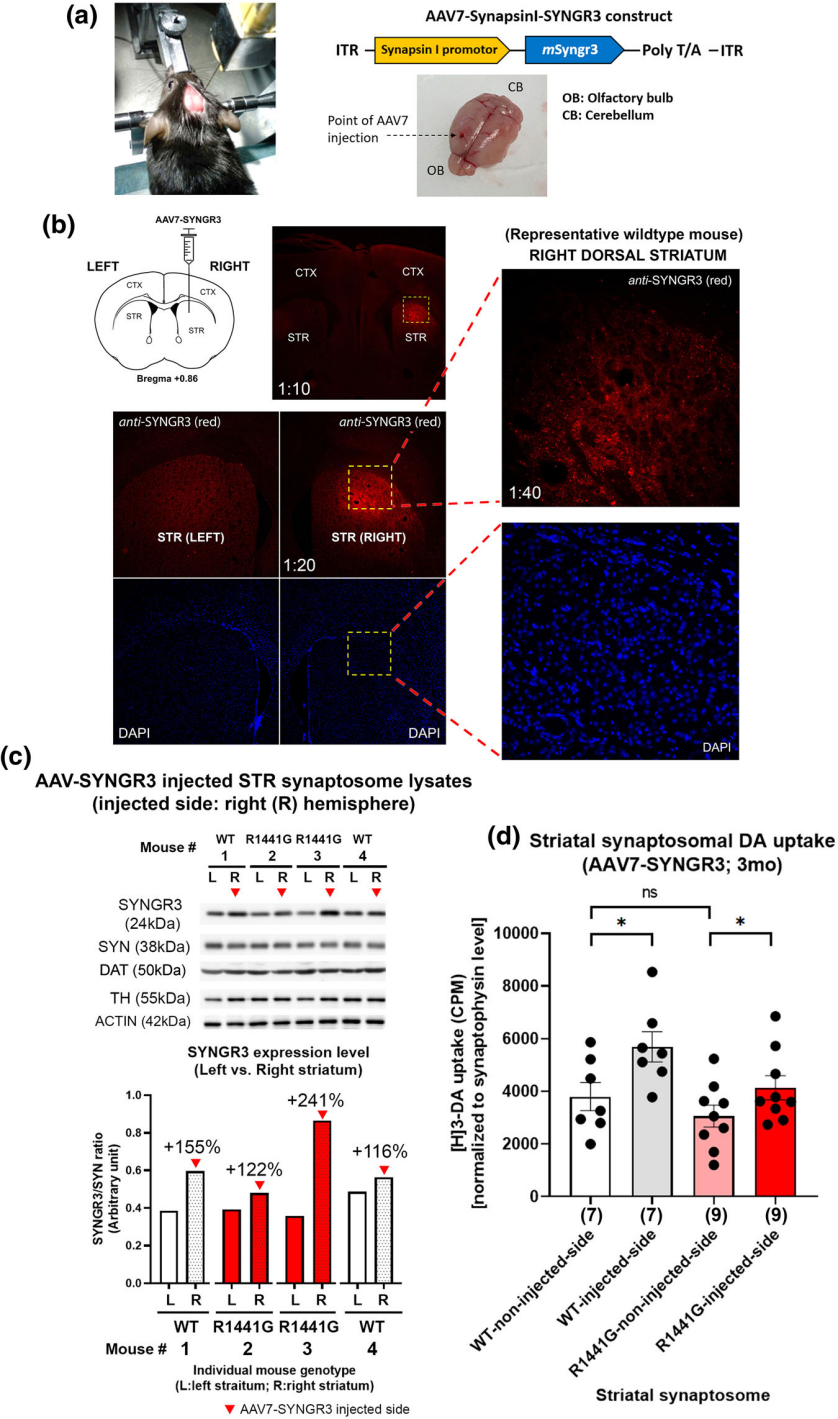
Adeno‐associated virus (AAV)‐mediated overexpression of SYNGR3 in striatum increased synaptosomal dopamine (DA) uptake. (a) Young 3‐month‐old wild‐type (WT) and LRRK2 mutant mice received intracranial injection of AAV7 (1 μl; injection rate: 0.1 μl/min) at one side of the brain hemisphere to induce SYNGR3 overexpression driven by Synapsin‐I promotor. WT and LRRK2 mutant mice were injected with AAV7‐SYNGR3 for 3 months before sacrifice. Diagram illustrates the design of the viral expression construct and the site of injection. (b) Overexpression of SYNGR3 on the injected side of the brain was confirmed by immunohistochemistry of SYNGR3 (red) compared to the opposite hemisphere of the same mouse brain. (c) Representative Western blots of two WT and two LRRK2^R1441G^ mutant mice showing overexpression of SYNGR3 in synaptosomal lysates extracted from injected mouse striatum 3 months after AAV injection. (d) Overexpression of SYNGR3 significantly increased striatal synaptosomal [3H]‐DA uptake, compared to the opposite non‐injected side of the striatum (paired *t*‐test). There was no significant difference in synaptosomal DA uptake at non‐injected side of WT and LRRK2 mutant striatum. *p < .05 represents statistical significance between designated groups by paired *t*‐test. Abbreviation: ns, not significant. R1441G, LRRK2^R1441G^ knockin mice

Having found that increasing SYNGR3 expression in SH‐SY5Y cells increased cellular DA uptake, we further explored whether inducing SYNGR3 expression in mouse striatum would have an effect on striatal synaptosomal DA uptake. Mice infected with endotoxin‐free adeno‐associated virus (AAV7)‐SynI‐*m*SYNGR3 were sacrificed 3 months after intracranial viral injection (1 μl viral suspension per hemisphere; injection rate: 0.1 μl/min) (Figure 4a). Injection titer of AAV7‐*m*SYNGR3 was 2.07 × 10^13^ VG/ml. Left and right striata of the brain were dissected separately and homogenized to isolate total synaptosomes for DA uptake assay and Western blot analyses. Compared to the non‐injected side of the same mouse brain, synaptosomes isolated from striatum injected with AAV7‐*m*SYNGR3 exhibited markedly higher levels of SYNGR3 expression, confirmed by immunohistochemistry (Figure 4b) and Western blotting of SYNGR3 (Figure 4c). Moreover, SYNGR3 expression level in the adjacent frontal cortex was not increased unlike their corresponding striatum injected with AAV, indicating that AAV7 diffused only within a limited range. SYNGR3 was overexpressed only in striatum as we had targeted (Figure S1).

Overexpression of SYNGR3 significantly increased striatal synaptosomal [3H]‐DA uptake, compared to the striatum on the opposite non‐injected side of the same brain (*p* < .05; N ≥ 7; paired Student's *t*‐test) (Figure 4d). DA uptake levels in each synaptosome isolate were normalized by their corresponding SYP level to ensure equal purity of synaptosome in each sample. There was no significant difference in synaptosomal DA uptake between the non‐injected sides of WT and LRRK2 mutant striata. These results indicate that induced SYNGR3 expression significantly increased striatal synaptosomal DA uptake.

### 3.5 In vivo overexpression of SYNGR3 in striatum alleviated impaired marble burying behavior in LRRK2 mutant mice

**FIGURE 5:**
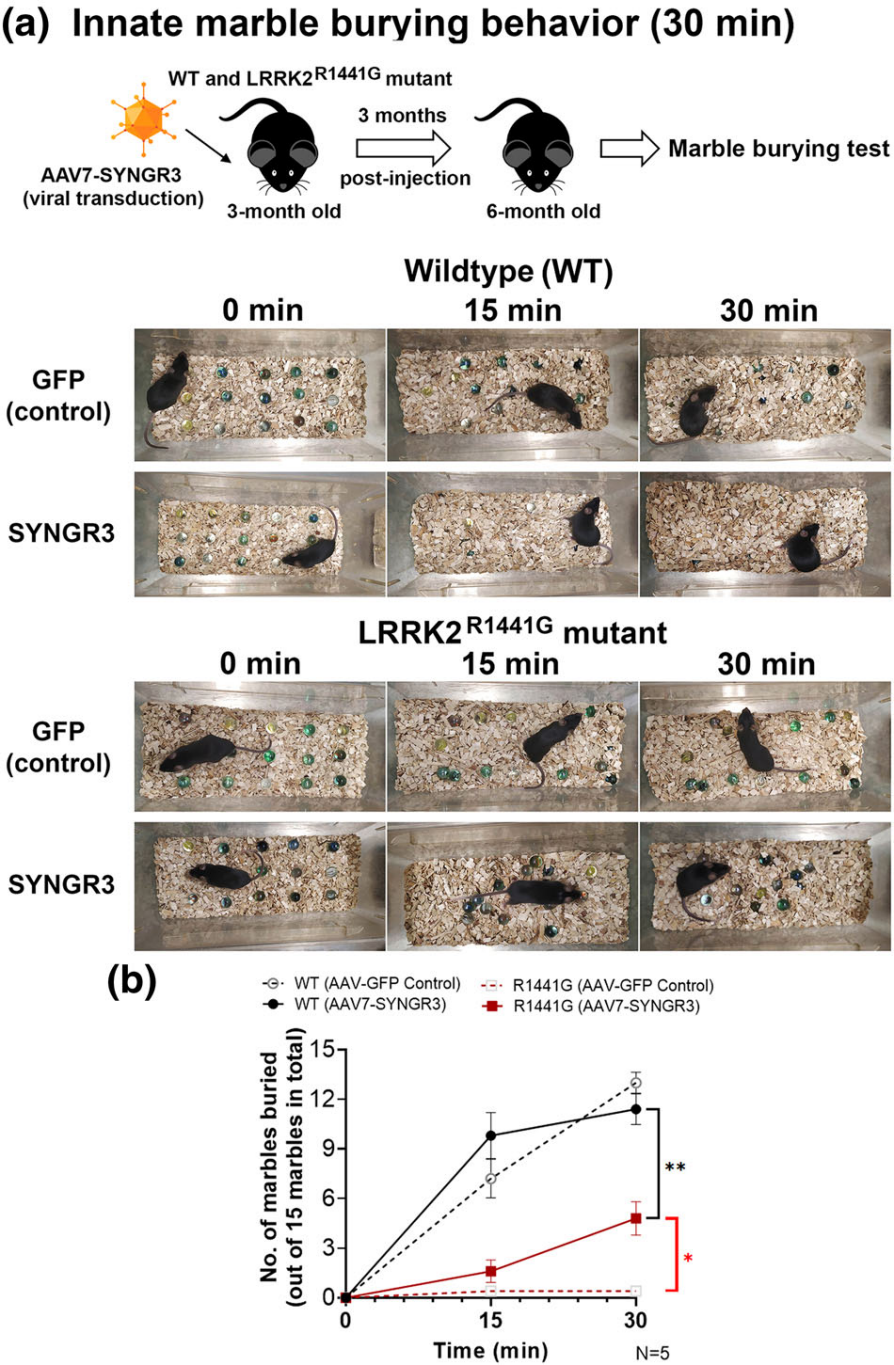
Effects of SYNGR3 expression on innate marble burying behavior. (a) Young (3 months old) wild‐type (WT) and LRRK2^R1441G^ mutant receiving stereotaxic injection of either adeno‐associated virus‐7–green fluorescent protein (AAV7–GFP) or AAV7‐SYNGR3 were subjected to marble burying test at 3 months after AAV injection. A total of 15 marbles were evenly distributed on the bedding inside the cage in form of a 5 × 3 matrix. (b) The number of marbles which have been covered two‐thirds by bedding were counted at 15 and 30 min. Total number of marbles buried (at 30 min) by LRRK2 mutant mice were significantly increased after overexpressing SYNGR3 in the striatum. Data are expressed as means ± SEM. (N = 5). *p < .05 and **p < .01 represent statistical significance between the two designated groups by unpaired, Student's *t*‐test.

As we found that overexpressing SYNGR3 in mouse striatum increased synaptosomal DA uptake activity, we further examined whether inducing SYNGR3 expression in the LRRK2 mutant brain would affect innate marble burying activity. Young (3‐month‐old) WT and LRRK2^R1441G^ mutant mice receiving stereotaxic injection of either AAV7‐*m*SYNGR3 or control AAV (AAV7‐GFP) for 3 months were subjected to marble burying test (Figure 5a). Our results showed that normal WT mice (at the age of 6 months) buried most of the marbles within 30 min of marble burying test (Figure 5b). However, age‐matched LRRK2 mutant mice failed to bury most of the marbles within the time period of the test compared to WT (*p* < .01; N = 5). Interestingly, LRRK2 mutant mice overexpressing SYNGR3 in the striatum exhibited a significant improvement in the total number of marbles buried, compared to their mutant counterparts injected with control AAV7‐GFP (*p* < .05; N = 5) (Figure 5b). Inducing SYNGR3 expression in WT mouse striatum caused no significant difference in the marble burying test performance (Figure 5b).

Furthermore, apparent aging effect in marble burying activity was seen in untreated WT and LRRK2 mutant mice. Both young (3‐month‐old) and aged (14‐month‐old) LRRK2 mutant mice buried significantly less marbles than their age‐matched WT mice (all *p* < .01) (Figure S2).

## 4 DISCUSSION

We reported significantly lower striatal SYNGR3 protein level in young LRRK2^R1441G^ mutant mouse model of PD (Liu et al., 2014). We also demonstrated an age‐dependent reduction in total SYNGR3 protein levels in the striatum of WT mice and a similar reduction of another vesicle marker protein, synaptophysin (SYP) in these animals, indicating reduced synaptic vesicles and/or nerve termini with aging in the CNS (Masliah et al., 1993; Petralia et al., 2014). Interestingly, this aging effect on SYNGR3 levels was not seen in LRRK2 mutant mice and was likely masked by the effect of LRRK2 mutation, which caused an early abnormal reduction of striatal SYNGR3 protein expression. These findings indicate that both aging and LRRK2 mutation affect striatal SYNGR3 expression. More importantly, throughout the life of young mutant compared to WT mice, low levels of SYNGR3 in mutant animals could chronically stress the DA turnover process and synaptic function, such that the process may eventually decompensate and lead to synaptic dysfunction and adverse pathological consequences with aging. How LRRK2 mutation affects SYNGR3 expression remains to be resolved, but it may involve early regulation of SYNGR3 gene expression in the young, for example, by a differential epigenetic regulation of gene promoter activity of SYNGR3 (Rivenbark et al., 2006). We recently carried out an in silico analysis of the 5′‐flanking region of *Syngr3* gene, where we identified CpG‐rich regions and key transcriptional regulatory elements including NBRE that binds to NURR1 protein, a key regulator in the development and survival of dopaminergic neurons (Li et al., 2022). Moreover, our findings mirror an earlier study showing that *SYNGR3* gene expression is age‐dependently decreased in healthy humans by about 24% over a period of 10 years (Saetre et al., 2011). A previous clinical neuroimaging study using positron emission tomography (PET) and 4‐hr‐long 18F‐fluorodopa (FD) scans showed a significant negative correlation between age and magnitude of decrease in effective DA distribution volume (EDV) in the putamen of PD brains (Sossi et al., 2006). In light of the role of SYNGR3 in DA neurons, reduced SYNGR3 level with age is consistent with epidemiological studies which report significantly increased incidence of synaptic dysfunction in the elderly with PD, implicating its potential pathogenic involvement. Similarly, endogenous expression of SYNGR3 gene and protein was also reduced in human post‐mortem brain tissues from patients with AD (Saetre et al., 2011; Williams et al., 2021; Wu et al., 2019). Therefore, it is plausible that reduced SYNGR3 expression may impair synaptic function in these age‐related neurodegenerative disorders.

Our previous studies showed that young LRRK2 mutant mice exhibited greater impairment of synaptosomal DA uptake in striatum after being stressed with VMAT‐2 inhibitor (reserpine) compared with WT controls (Liu et al., 2014). These mice recovered more slowly from reserpine‐induced locomotor deficits compared to their age‐matched WT littermates (Liu et al., 2014), indicating greater susceptibility to compromised DA turnover. We also previously showed that inherent V‐ATPase levels in mutant mouse striatal synaptosomal lysates were significantly lower, which contributes to the increased susceptibility to rotenone‐induced DA depletion in these LRRK2 mutant animals (Liu et al., 2017). Here, we continued to explore whether modulating SYNGR3 expression could facilitate striatal DA uptake in young LRRK2 mutant mice. We first determined whether increasing SYNGR3 expression had any influence on cellular DA uptake activity using human neuronal cell culture in vitro. Differentiated SH‐SY5Y cells have catecholaminergic neuronal features such as expression of TH and DAT, but they express low levels of SYNGR3 (Kovalevich & Langford, 2013; Xicoy et al., 2017). Therefore, we transiently overexpressed SYNGR3 in SH‐SY5Y cells and compared them with control cells transfected with an empty expression vector. These engineered cells expressed SYNGR3 protein at more than a 200‐fold level compared with the control cells. Immunohistochemistry showed that the SYNGR3 was expressed in close proximity to another marker protein, SYP, expressed on vesicles, indicating that SYNGR3 was localized at the appropriate subcellular compartment. We showed that overexpression of SYNGR3 increased whole‐cell DA uptake by >20%. This is a physiologically significant increase compared with the uptake seen in control cells which expressed endogenous SYNGR3 at relatively lower levels (Prasad & Amara, 2001). Western analysis showed that SYNGR3 overexpression did not affect DAT level, confirming that the increase of DA uptake was not due to increased DAT levels. These findings mirrored similar results in an earlier study by Egana et al. (2009) where SYNGR3 expression increased DA uptake in two mouse cell lines, MN9D and PC12. This earlier study also showed that the effect of SYNGR3 on DAT function was abolished by reserpine (VMAT2 inhibitor), suggesting that this interaction may contribute to the sequestration of DA into vesicles via VMAT‐2 (Egana et al., 2009).

**FIGURE 6:**
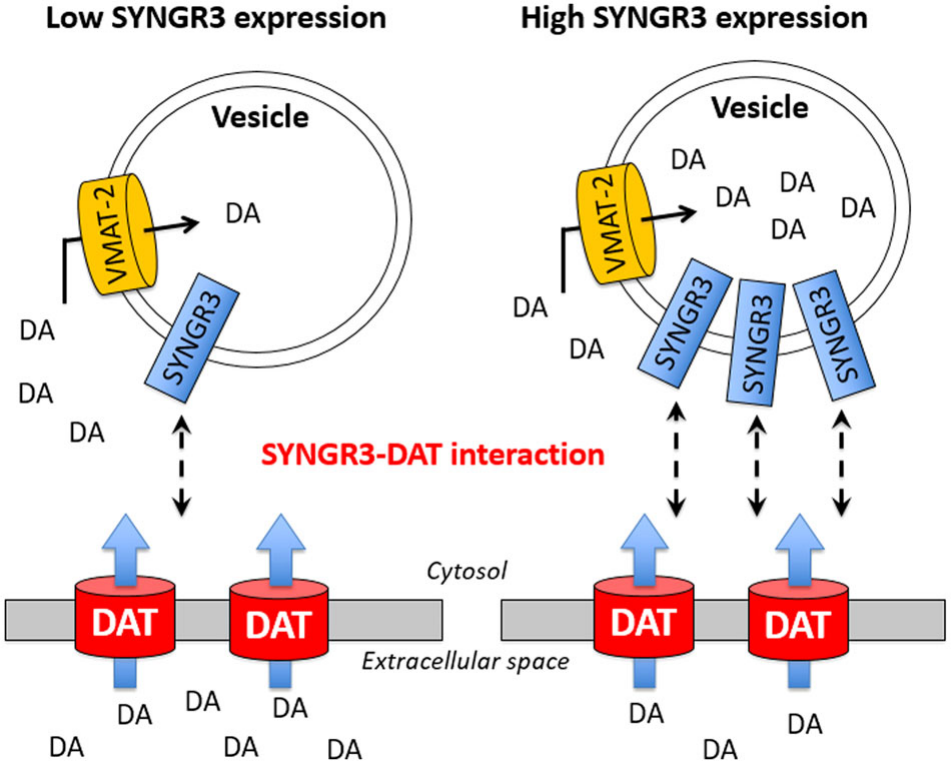
Schematic diagram illustrating how SYNGR3 expression level can affect dopamine (DA) uptake and sequestration into synaptic vesicles via interaction between SYNGR3 and DAT. Egana et al. (2009) first reported interaction between SYNGR3 and DAT in the vesicular DA storage system. Induced expression of SYNGR3 on synaptic vesicle surface can recruit vesicles into close proximity to dopamine uptake transporter (DAT) near the plasma membrane for more efficient and rapid sequestration of cytosolic free DA into synaptic vesicles via VMAT‐2.

Having confirmed the effects of SYNGR3 on DA uptake in vitro, we further explored the relationship of SYNGR3 to DAT in vivo. DAT activity is regulated by complex mechanisms that modulate clearance of extracellular DA in response to physiological conditions (Bu et al., 2021). In particular, interactions between DAT and its ligands have been shown to promote the formation of complexes involved in DA turnover (Vaughan & Foster, 2013). We showed by immunohistochemistry and immuno‐gold TEM that SYNGR3 co‐localized with DAT in the striatal pre‐synaptic terminals. We also demonstrated by co‐immunoprecipitation that in extracts from the mouse striatum, SYNGR3 physically bound to DAT. Although our TEM images do not completely illustrate the gross structure of synaptic terminals due to technical limitations of tissue fixation which can compromise immunostaining efficiency, relevant structural components were clearly identified, including the pre‐synaptic terminals (the more electron‐dense region) and vesicles (circular sac‐like structures). The immuno‐gold TEM images showed a portion of the total SYNGR3 which co‐localized with DAT on any given snapshot. This is not surprising because not all SYNGR3 proteins need to be bound permanently to DAT given its potential function to facilitate the dynamic process of vesicular transport in pre‐synaptic nerve terminals. Synaptic vesicles differ in their availability for release and mobilization in response to different stimuli that determine synaptic strength and plasticity (Alabi & Tsien, 2012). SYNGR3 level was reduced in PD brains (Simunovic et al., 2009). The importance of attenuating SYNGR3 deficiency is to strengthen its interaction with DAT which subsequently bring these vesicles into close proximity to DAT at the plasma membrane. This spatial arrangement would facilitate more efficient sequestration of cytosolic free DA into synaptic vesicles via VMAT‐2 (Figure 6). This has an important physiological implication in minimizing the risk of auto‐oxidation of free DA and oxidative stress at the synaptic terminal (Herrera et al., 2017).

Our earlier study found that LRRK2^R1441G^ mutant mice had a significant age‐dependent decrease in striatal synaptosomal DA uptake (Liu et al., 2014). In this study, we found that the lower SYNGR3 level in young mutant mice did not result in reduced striatal DA uptake. This may be due to compensatory mechanisms, such as a more rapid vesicle trafficking or recycling despite a lower SYNGR3 level in young mutant mice (Croft et al., 2005), thus maintaining synaptic DA turnover and function. This could possibly explain why young LRRK2^R1441G^ mutant mice do not show obvious motor phenotypes without being stressed. Yet, this energy‐consuming process to hasten vesicle recycling could become a lifetime stress factor contributing to early synaptic dysfunction and DA cell death. Our results are in accord with previous work on the involvement of LRRK2 in presynaptic function, where mutant LRRK2 was shown to perturb vesicular trafficking and spatial distribution in the pre‐synaptic pool (Piccoli et al., 2011). Nevertheless, the molecular links between LRRK2 and other synaptic factors are unclear but is thought to involve modulation of LRRK2 macro‐molecular complexes with synaptic proteins (Cirnaru et al., 2014), such as N‐ethylmaleimide‐sensitive factor, adaptor protein 2 complex subunits, synaptic vesicle protein 2A, synapsin 1A, syntaxin 1, dynamin‐1, clathrin (Piccoli et al., 2011), Rab5b (Shin et al., 2008), and actin (Meixner et al., 2011). Whether SYNGR3 interacts with LRRK2 in a similar manner as the other synaptic proteins requires further investigation.

Given that LRRK2 mutant mice are prone to striatal DA depletion (Liu et al., 2014), we explored whether in vivo induction of SYNGR3 expression in mutant brain can promote striatal DAT activity. Simultaneously, we performed a preliminary assessment of animal innate behavior using marble burying test. Viral‐based gene delivery is a well‐developed experimental approach to overexpress the target protein in mouse brains (Royo et al., 2007). Young WT and LRRK2 mutant mice injected with AAV for 3 months demonstrated significant overexpression of SYNGR3. The ipsilateral striatum with SYNGR3 overexpression demonstrated significantly higher synaptosomal DA uptake activity compared to the control contralateral striatum expressing SYNGR3 at the physiological level. Moreover, increased SYNGR3 expression in the treated ipsilateral striatum was not associated with significant changes in SYP levels, indicating an absolute increase in SYNGR3 amount relative to vesicles rather than an increase in the total vesicles number after SYNGR3 overexpression. This is crucial because the increased SYNGR3 on the surface of each vesicle can presumably induce stronger interaction with DAT. This may explain why increased expression of SYNGR3 in mouse striatum resulted in increased striatal DA uptake activity.

DA neurons project from SNpc and ventral tegmental area (VTA) to modulate neuronal activity in the striatal complex including the caudate putamen (CPu) and nucleus accumbens (NAc), which collectively regulate a variety of motor and non‐motor function implicated in PD. DA levels in dorsomedial striatum have been shown to correlate with innate and inflexible behavior in human (Haber et al., 2000; Knowlton et al., 1996; van Elzelingen et al., 2022). Here, we explored whether an increase in striatal DA uptake via inducing SYNGR3 expression in LRRK2 mutant mice could influence their innate behavior. Marble burying test has been used to assess innate behavior in rodents (Himanshu et al., 2020; Nicolas et al., 2006; Njung'e & Handley, 1991). The number of marbles buried by the testing mice within a certain time period as a result of innate digging behavior can serve as an objective quantitative measure of this behavior given that the comparisons are performed under similar environmental conditions such as light intensity, temperature, scent, and bedding (Himanshu et al., 2020). Interestingly, we found that young LRRK2 mutant mice failed to bury most of the marbles within the time period of the test (i.e., 30 min), in contrast to WT mice which buried most of the marbles within 15 min. These findings suggest that LRRK2 mutation was associated with a significant impairment of this innate marble burying behavior. Similar to the age‐dependent reduction of striatal SYNGR3 expression, a greater apparent aging effect in marble burying activity was also observed in WT mice compared to mutant mice due to the fact that young mutant mice had already significantly impaired marble burying activity (Figure S2). Although the molecular mechanism which regulates such innate behavior could be multifactorial and complex (Grant, 2018; Njung'e & Handley, 1991; Sugimoto et al., 2007), both young and aged LRRK2 mutant mice buried significantly less marbles than their age‐matched WT mice, indicating behavioral impairment associated with LRRK2 mutation. Furthermore, young LRRK2 mutant mice which had SYNGR3 overexpression from the AAV injection demonstrated a significant improvement in their marble burying activity compared to mutant littermate controls which received the sham surgical procedure. These findings indicate that induced SYNGR3 expression in striatum which promoted DA uptake could alleviate impaired marble burying behavior in LRRK2 mutant mice. Nevertheless, based on our limited understanding, we cannot exclude other possibilities to explain this preliminary behavioral observation. A limitation to the marble burying test is that apart from burying alien objects such as marbles, rodents may show other behavior changes such as burrowing, rearing, and grooming (Deacon & Rawlins, 2006), which were not assessed in this study. While this test has been used to study the effects of various drugs during therapeutic trials on experimental rodent models of different human disorders including anxiety and obsessive‐compulsive behavior (Nicolas et al., 2006; Njung'e & Handley, 1991), it is yet unclear how specific this test is in assessing a certain behavior (Gyertyán, 1995; Londei et al., 1998; Thomas et al., 2009). Nevertheless, apart from DA, 5‐HT_2A_ receptor agonists were shown to inhibit digging behavior in marble burying test (Lim et al., 2018). Thus, there are technical limitations to devise a test that is exclusively indicative of dopamine activity. Previous study showed that LRRK2^G2019S^ mutation induced anxiety or depression behavior in mice via upregulation of 5‐HT_1A_ receptor (Odland et al., 2021). Whether LRRK2^R1441G^ mutation is causing anxiety and depression and why this mutation impaired marble burying behavior require further investigation.

There are recent studies which demonstrated that SYNGR3 mediated pathological tau protein recruitment to the pre‐synapse in an AD mouse model by facilitating tau binding to synaptic vesicle membrane (McInnes et al., 2018). These studies indicated that lowering SYNGR3 expression could attenuated tau‐induced memory defects and synaptic loss in mice. Although the findings appear to have different implications on the effects of increasing SYNGR3 expression in brain, they are not mutually exclusive to our current study. The objectives and indicators of efficacy in distinctly separate experimental mouse models of AD and PD are clearly different.

In conclusion, using LRRK2^R1441G^ knockin mice that we have previously shown to be more susceptible to striatal DA depletion, SYNGR3 protein expression in young mutant mouse striatum was significantly lower compared to age‐matched WT mice. We demonstrated co‐localization and interaction between SYNGR3 and DAT, a key player in synaptic DA uptake and turnover, implicating their roles in facilitating synaptic function in the nigrostriatal neural network. Overexpression of SYNGR3 increased cellular DA uptake in human neuronal cells and in mouse striatum ex vivo. Furthermore, LRRK2 mutant mice overexpressing SYNGR3 by AAV transduction in the striatum demonstrated significant alleviation of impaired innate marble burying behavior associated with a concomitant elevation of striatal synaptosomal DA uptake. Our findings with a preliminary behavioral assessment advocate further studies to determine the influence of SYNGR3 in the pathophysiology of DA neurons in PD.

## AUTHOR CONTRIBUTIONS

Philip Wing‐Lok Ho, Shu‐Leong Ho, and David Boyer Ramsden designed the project. Lingfei Li, Philip Wing‐Lok Ho, Eunice Eun Seo Chang, Chi‐Ting Leung, Hui‐Fang Liu, Yasine Malki, and Zoe Yuen‐Kiu Choi performed experiments and data analysis. Chi‐Ting Leung performed tissue sectioning, confocal imaging, and related analyses. Michelle Hiu‐Wai Kung managed routine laboratory activities and safety. Shu‐Leong Ho, Philip Wing‐Lok Ho, and David Boyer Ramsden oversaw and evaluated all experimental data. Philip Wing‐Lok Ho, Shirley Yin‐Yu Pang, David Boyer Ramsden, and Shu‐Leong Ho wrote the manuscript. All authors read and approved the final manuscript.

## CONFLICTS OF INTEREST

The authors declare no conflict of interest.

### PEER REVIEW

The peer review history for this article is available at https://publons.com/publon/10.1002/brb3.2886

## Supporting information

Supplementary Material
Supplementary Figure S1. SYNGR3 levels in adjacent frontal cortex from AAV7‐*m*SYNGR3 injected mice were not increased as in their corresponding striatum after AAV injection. Top panel showed representative Western blots of SYNGR3 in adjacent frontal cortex extracted from 2 WT and 2 mutant (R1441G) mice 3 months after AAV injection. “ns”: not significant.
Supplementary Figure S2. Marble burying activity of young (3‐month‐old) and aged (14‐month‐old) WT and LRRK2 mutant mice. A total of 15 marbles were evenly distributed on the bedding inside the cage in form of a 5 x 3 matrix. The number of marbles which have been covered 2/3 by bedding were counted at 15 and 30 min. Total number of marbles buried by both young and aged LRRK2 mutant mice were significantly lower than their age‐matched WT mice. Data are expressed as means ± SEM. ** *p* < .01 represents statistical significance as compared to WT mice at their corresponding time point by unpaired, Student's t‐test.

## Data Availability

The original contributions presented in the study are included in the article/supplementary material, further inquiries can be directed to the corresponding authors.
