# Supplementary material for: In vivo overexpression of synaptogyrin‐3 promotes striatal synaptic dopamine uptake in LRRK2R1441G mutant mouse model of Parkinson's disease
Source: Brain Behav. 2023 Jan 9;13(2):e2886. doi: 10.1002/brb3.2886 (PMC9927849; doi:10.1002/brb3.2886)
Supplement: Supplementary file 1 — Supplementary Material Supplementary Figure S1. SYNGR3 levels in adjacent frontal cortex from AAV7‐mSYNGR3 injected mice were not increased as in their corresponding striatum after AAV injection. Top panel showed representative Western blots of SYNGR3 in adjacent frontal cortex extracted from 2 WT and 2 mutant (R1441G) mice 3 months after AAV injection. “ns”: not significant. Supplementary Figure S2. Marble burying activity of young (3‐month‐old) and aged (14‐month‐old) WT and LRRK2 mutant mice. A total of 15 marbles were evenly distributed on the bedding inside the cage in form of a 5 x 3 matrix. The number of marbles which have been covered 2/3 by bedding were counted at 15 and 30 min. Total number of marbles buried by both young and aged LRRK2 mutant mice were significantly lower than their age‐matched WT mice. Data are expressed as means ± SEM. ** p < .01 represents statistical significance as compared to WT mice at their corresponding time point by unpaired, Student's t‐test. [file BRB3-13-e2886-s001.docx]

Supplementary Material

**Supplementary Figures and legends:**


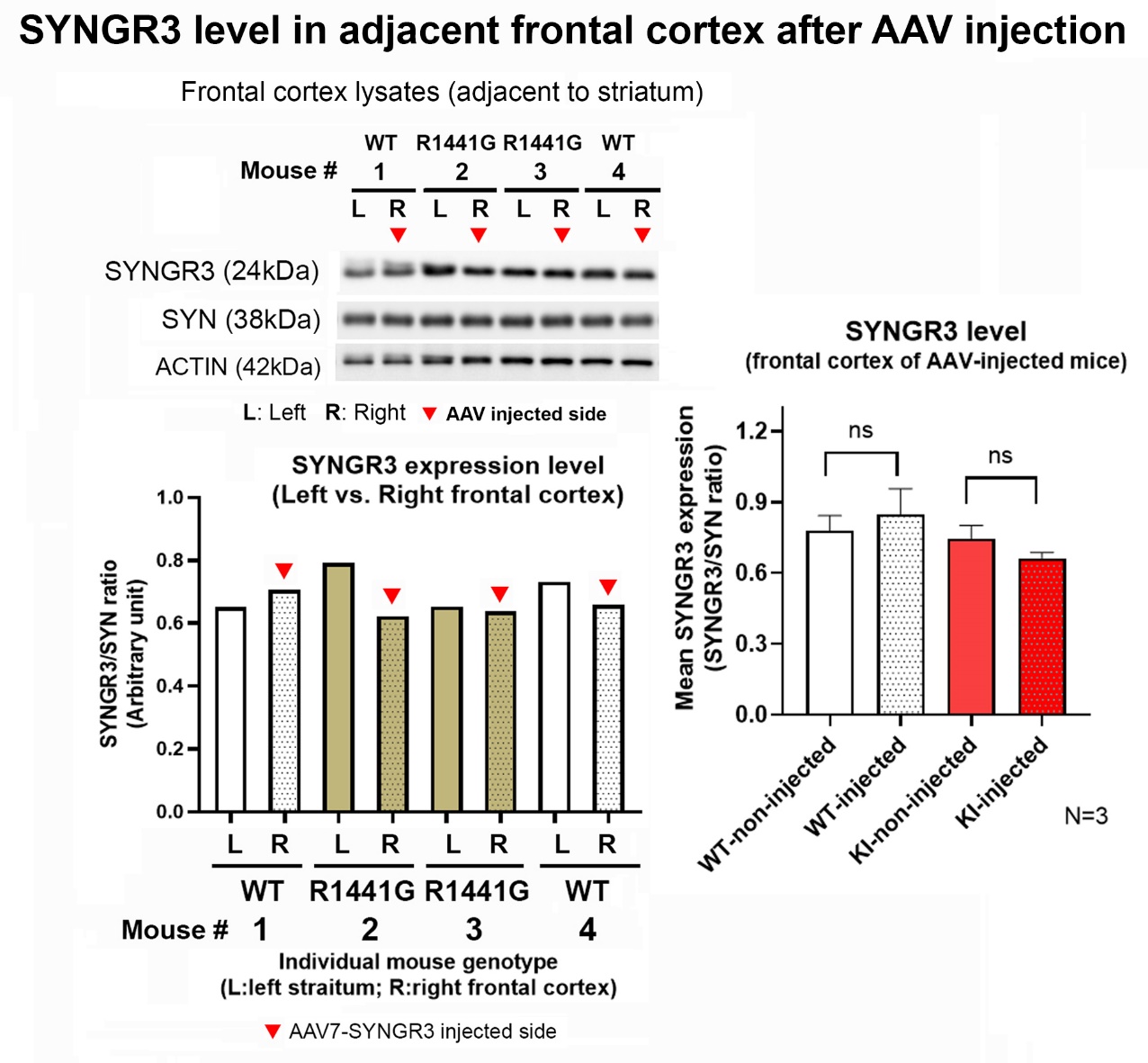


**Supplementary Figure S1.** SYNGR3 levels in adjacent frontal cortex from AAV7-*m*SYNGR3 injected mice were not increased as in their corresponding striatum after AAV injection. Top panel showed representative Western blots of SYNGR3 in adjacent frontal cortex extracted from 2 WT and 2 mutant (R1441G) mice 3 months after AAV injection. “ns”: not significant.


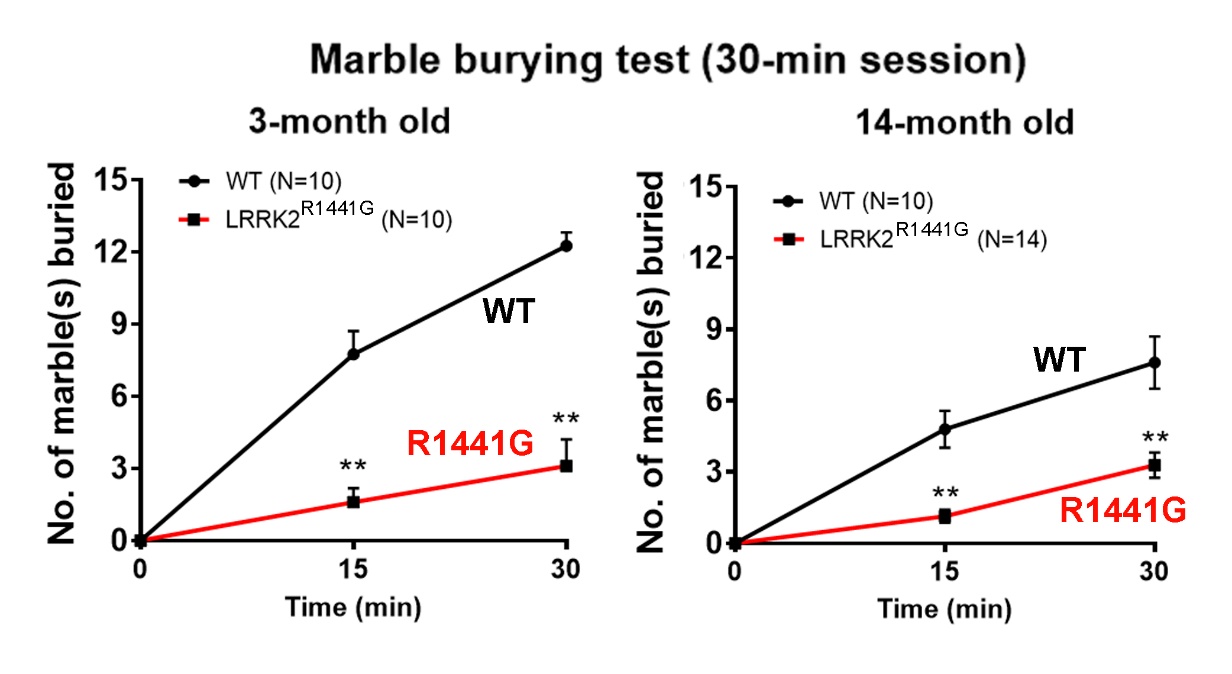


**Supplementary Figure S2.** Marble burying activity of young (3-month old) and aged (14-month old) WT and LRRK2 mutant mice. A total of 15 marbles were evenly distributed on the bedding inside the cage in form of a 5 x 3 matrix. The number of marbles which have been covered 2/3 by bedding were counted at 15 and 30 min. Total number of marbles buried by both young and aged LRRK2 mutant mice were significantly lower than their age-matched WT mice. Data are expressed as means ± SEM. ** p<0.01 represents statistical significance as compared to WT mice at their corresponding time point by unpaired, Student’s t-test.
